# Transfusion-induced *Plasmodium falciparum* malaria in a beta thalassaemia patient during the prevention of re-establishment phase in Sri Lanka

**DOI:** 10.1186/s12936-021-03881-1

**Published:** 2021-08-26

**Authors:** Pubudu Chulasiri, Prasad Ranaweera, Ponnuthurai Sudarshan, Maya Jayasinghe, Jeevani Harishchandra, Kumudu Gunasekera, Harshini Vitharana, Priyanganie Silva, Pascal Ringwald, Rohini Fernandopulle, Kamini Mendis, Deepika Fernando

**Affiliations:** 1 Anti Malaria Campaign, Ministry of Health, Colombo, Sri Lanka; 2 District General Hospital, Polonnaruwa, Sri Lanka; 3 Regional Malaria Office, Polonnaruwa, Sri Lanka; 4 Global Malaria Programme, World Health Organization, Geneva, Switzerland; 5 Faculty of Medicine, General Sir John Kotelawala Defense University, Ratmalana, Sri Lanka; 6 Department of Parasitology, Faculty of Medicine, University of Colombo, Colombo, Sri Lanka

**Keywords:** Malaria, Blood transfusion, Prevention of re-establishment, Induced malaria

## Abstract

**Background:**

Malaria was eliminated from Sri Lanka in 2012, and since then 50–60 imported malaria cases have been reported yearly. The country has remained malaria-free since, except for a single case of indigenous malaria in 2018. Blood donors are routinely screened for malaria, and transfusion malaria has not been reported in the country since 1966.

**Case presentation:**

A 17-year-old splenectomized beta thalassaemia patient developed a transfusion-induced *Plasmodium falciparum* malaria infection following a blood transfusion 18 days earlier. The blood donor was an armed forces personnel who returned from South Sudan following a United Nations peace-keeping mission. The blood recipient’s malaria infection took a complicated clinical course with elevated liver enzymes, lowered blood pressure and a prolonged parasite clearance time of 7 days but he recovered fully after two courses of artemether-lumefantrine interrupted by a course of intravenous artesunate. The prolonged parasite clearance is likely due to lack of splenic clearance of dead or damaged intra-erythrocytic parasites (due to a splenectomy) rather than to the parasite strain being resistant to artemisinin or the partner drug. This is corroborated by the fact that the blood donor’s infection responded to artemether-lumefantrine with parasites being cleared on day 3. The blood donor who had not displayed signs or symptoms of malaria, had been screened for malaria on arrival in Sri Lanka and was negative on both microscopy and RDT. At the point of blood donation a blood smear examined microscopically was also reported negative for malaria, but retrospectively, the preserved smear of the donor’s blood was found to contain *P. falciparum* parasites at a very low density. The donor when tested after the transfusion-induced case was diagnosed, also tested positive for malaria and was treated.

**Conclusions:**

After malaria elimination, transfusion-induced malaria from blood donors returning from malaria endemic countries poses a threat to preventing the re-establishment of the disease. Improved surveillance of arrivals in Sri Lanka from malaria endemic countries using more sensitive methods for screening than microscopy may be required to reduce this risk. More stringent criteria for selecting blood donors, and more effective methods of screening donors for malaria than microscopy may also be necessary.

**Supplementary Information:**

The online version contains supplementary material available at 10.1186/s12936-021-03881-1.

## Background

Since being certified by the World Health Organization (WHO) as a malaria-free country in 2016, Sri Lanka reports 50–60 imported malaria cases a year [1]. Except for a single case of indigenous malaria reported in 2018, the country has been maintained free of malaria transmission, despite a high receptivity due to the presence of malaria vectors. This has been possible due to stringent malaria case surveillance by passive and active case detection and entomological surveillance and response programmes. In addition to this, screening of blood donors for malaria is carried out by microscopy by the Anti Malaria Campaign (AMC), the national malaria programme within the Ministry of Health of Sri Lanka and is also an integral part of malaria surveillance. Of approximately one million blood smears screened each year for the presence of malaria parasites, nearly 40% are those of blood donors to minimizes the risks of transfusion-induced malaria [2]. A further aspect of haemovigilance rests on the policy of barring candidates from blood donation for 3 months following their return from a malaria endemic country.

Sri Lanka has reported 16 transfusion-induced malaria cases between 1960 and 1966 when malaria was endemic in the country leading to 14 *Plasmodium malariae* and two *Plasmodium falciparum* infections [3], but none since then. The case presented here constitutes the first blood transfusion-induced case of malaria in over 5 decades and has implications for the prevention of re-establishment of malaria.

## Case presentation

### The blood recipient

The recipient of the blood transfusion was a 16-year-old Sri Lankan boy who has beta thalassaemia major with a co-morbidity of blood transfusion-induced cardiomyopathy. Thalassaemia is the commonest monogenic disease in Sri Lanka [4]. The patient, who is from the Polonnaruwa district in the North Central Province of Sri Lanka gave no history of travel overseas. He had been splenectomized in 2010 and receives monthly blood transfusions. His last transfusion had been on the 21st of April 2021. He developed fever and headache 13 days following the transfusion on 4th May for which he sought treatment from a General Practitioner two days later. He was treated for a viral fever with anti-pyretics and malaria was not suspected nor tested for at this stage of the illness. As the fever did not respond to treatment he was admitted to the General Hospital, Polonnaruwa on the 9th of May 2021 for further investigation and treatment, 18 days after the blood transfusion. As a part of the routine fever surveillance activities carried out in hospitals by the Public Health Field Officer (PHFO) for the AMC, the patient was tested for malaria soon after admission, and was reported positive for *P. falciparum* by microscopy (parasite density of 51,315/μl with asexual parasites and gametocytes). The diagnosis was also confirmed by polymerase chain reaction testing.

The day after admission, when the malaria diagnosis was confirmed the patient was febrile (37.8 °C), had a blood pressure of 90/60 Hgmm and a normal heart rate. Laboratory investigations revealed a haemoglobin (Hb) of 5.4 mg/dl, WBC count of 31,300/μl and a platelet count of 378,000/mm^3^. The patient was treated with artemisinin-based combination therapy (ACT), artemether-lumefantrine, the first-line treatment for *P. falciparum* malaria in Sri Lanka, for 3 days at the standard recommended dosage under supervision, based on national malaria treatment guidelines [5]. Routine investigations on liver and other functions were performed.

The patient’s clinical condition deteriorated over the first 3 days of treatment. The peripheral blood parasitaemia declined very slowly from a starting density of 51,015 parasites/μl on admission. After 72 h of commencement of anti-malarial treatment the parasitaemia was 7147 parasites/μl, blood pressure remained below normal at 90/50 mmHg, haemoglobin (Hb) level fell to 7.6 mg/dl and the WBC count was 47,430/mm^3^. He still had irregular spikes of fever. The liver enzymes increased (AST from 86.5 U/L to 250.8 U/L and to 458.9 U/L; and ALT from 62 U/L to 174.3 U/L and 314.9 U/L) over 3 days. Serum C-Reactive Protein was elevated above 100 mg/L. The patient was managed with intravenous inotropes (noradrenalin) because of the poor response of blood pressure to fluid therapy. With parasites persisting in peripheral blood on completion of the ACT course at 72 h, and deterioration of the patient’s clinical condition, further anti-malarial treatment options were considered. The second-line anti-malarial medicine in Sri Lanka, dihydroartemisinin-piperaquine (DHAPPQ) could not be used in this patient because it is contraindicated in cardiomyopathies which the patient suffered from. Treatment with the next option, intravenous artesunate 2.4 mg/kg was, therefore, commenced immediately (i.e. 72 h after starting anti-malarials) and given for a further 3 days while the patient was managed in the Coronary Care Unit of the hospital. Following the last dose of IV artesunate, he was started on a 3-day course of oral artemether-lumefantrine as the recommended practice after parenteral artesunate. By day seven of commencement of treatment, asexual malaria parasites were no longer seen in blood smears, but gametocytes (sexual stages) were present at a density of 270/μl. The patient had improved clinically by then and had a stable blood pressure after withdrawal of inotropes, and liver function test had returned to normal levels (AST- 48.7 U/L and ALT- 25.5 U/L). The gametocyte count decreased gradually to 152 parasites/µl on completion of oral ACT on day 9. A stat dose of primaquine (0.75 mg/ kg^−3^ tablets) was given on day 10 for its anti-gametocyte activity. The patient was completely cleared of parasites including gametocytes by day 15. Following the transfusion of three units of blood the patient was discharged from hospital on day seventeen (post transfusion Hb was 9.8 mg/dl (27th April 2021). Data on beta thalassaemia patient diagnosed with malaria is provided in Additional file 1: Table S1.

### The blood donor

**Fig. 1:**
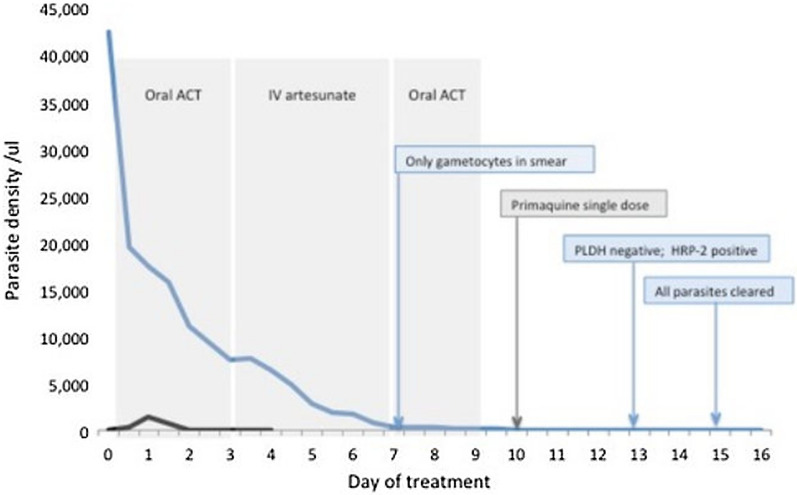
Parasitaemia, treatment schedule and diagnostic milestones of the donor and recipient. Clearance of parasitaemia in the blood recipient (blue line) and the donor (black line), and as call outs the treatment schedule (grey) and diagnostic milestones (blue) as they apply to the blood recipient. Only the first artemisinin-based combination treatment applies to the blood donor

Upon diagnosis of malaria in the recipient, information on the blood donor of the last transfusion given to the recipient on the 21st of April 2021 was traced, based on the records maintained by the National Blood Transfusion Service. The donor was a member of the armed forces who had returned to Sri Lanka on the 9th of December 2020 after spending 16 months in South Sudan on a United Nations peacekeeping mission. He was also traced in the AMC database as a person who was being kept under surveillance. While in South Sudan he had taken mefloquine and doxycycline as antimalarial prophylaxis, but reportedly, not on a regular basis. As per guidelines of the AMC, and through the close collaboration that exists with the armed forces, their members arriving from malaria endemic countries are screened for malaria by microscopy and Rapid Diagnostic Tests (RDT) at the airport, or, due to the current COVID-19 pandemic, at COVID quarantine centers where they are kept for 14 days. The first malaria screening of the group of 51 armed forces personnel of whom the blood donor was one, was performed at the airport on the 9th of December 2020 and he and the rest of the group were reported as negative for malaria by microscopy and RDT. He was asymptomatic on arrival and gave no history of malaria while in South Sudan. He had donated blood four months after his return, on the 9th of April 2021 and this blood donation was used on the beta thalassaemia patient. Prior to transfusion the blood product was screened for malaria by microscopy as a routine procedure but was reported as negative. However, when the stored blood smear was examined retrospectively after the incident of transfusion-malaria, it showed *P. falciparum* parasites (asexual parasites—112 parasites/μl, gametocytes—32 parasites/μl). On testing the asymptomatic donor for malaria on 10th May 2021, soon after the recipient was diagnosed, he was found to be positive for malaria by microscopy at a very low density of 32 parasites/ μl with ring and gametocyte stages of *P. falciparum*. On admission his Hb was 15.2 g/dl. He was admitted to the same hospital ward as the blood recipient a day later, and was treated with an oral ACT artemether-lumefantrine and a single dose of primaquine [5]. His blood parasitaemia was completely cleared on day 3 of treatment (Fig. 1) and was discharged from hospital on the 4th day.

### Malaria reactive surveillance

Reactive parasitological and entomological surveillance activities commenced the same day as the diagnosis of malaria was made in the blood recipient and donor respectively in accordance with AMC’s prevention of re-establishment strategy. Primary and secondary case surveillance was carried out covering all residents of houses within a radius of 1 km of residencies of both the recipient and the donor [6]. A total of two hundred persons were screened by microscopy and were found negative for malaria. The blood donor had 51 travel contacts in the armed forces group which had returned with him from South Sudan who were screened by microscopy, and they all reported negative for malaria.

Entomological surveillance was conducted within a radius of 1 km of the residence of the blood recipient. With the reporting of larvae of *Anopheles culicifacies*, the primary vector of malaria in Sri Lanka, larval source management, space spraying and distribution of long-lasting insecticidal nets (LLINs) were carried out. Entomological surveillance was also carried out around all sites where, the blood donor had stayed night over the past 14 days from blood donation up to diagnosis, and larvae of primary vector was found in two locations. The same vector control methods mentioned above were applied at these sites as well.

## Discussion

This is the first case of transfusion-induced malaria reported in Sri Lanka in five decades, and the first during the prevention of reintroduction (POR) phase of malaria. The recipient, who suffered from beta thalassaemia major and gave no history of overseas travel but of receiving a recent blood transfusion, was diagnosed with *P. falciparum* malaria. This was established as a case of blood transfusion-induced malaria by the fact that his last transfusion of blood was found to be infected with *P. falciparum*. The blood donor was a returnee from South Sudan having been on a mission there for a year and four months. Although microscopy at the time of blood donation was reported as malaria-negative, retrospective re-examination of the donor’s blood smear at the time of donation, as well as when he was tested after the recipient was diagnosed with malaria revealed that he had a *P. falciparum* malaria infection throughout, although he had remained free of signs and symptoms of malaria ever since he returned to Sri Lanka.

The beta thalassaemia patient had a clinically complicated malaria infection with co-morbidities, and prolonged parasite clearance. The possibility that this malaria infection imported from South Sudan was a drug-resistant strain, or even one with a reduced sensitivity to artemisinin or the partner drug lumefantrine was considered, but was dismissed on the grounds that the infection in the blood donor responded well, both clinically and parasitologically to the same medication. The prolonged parasite clearance is likely, instead, to have been due to the absence of the spleen. Such slow responses of malaria infections to treatment have been reported previously in splenectomized patients [7–9]. Intravenous artesunate was given to this patient following a full course of oral artemether-lumefantrine because of the persistent parasitaemia and deteriorating clinical condition, and also because the second-line medicine DHAPPQ was contraindicated on account of a cardiomyopathy he suffered from. The long duration of anti-malarial treatment with artemether-lumefantrine and intravenous artesunate resulted in eventual clearance of asexual parasites on day 7, and gametocytes on day 15, the latter in response to the single dose of primaquine.

Delays in the diagnosis of malaria has been a frequent feature during the POR phase in Sri Lanka [10, 11], as is also common in most countries where malaria is no longer endemic [12, 13]. However, in this patient malaria was diagnosed within 3 days of his seeking health care and soon after admission to hospital. The malaria infection was missed by the General Practitioner who was the patient’s first contact with the health system. However, on admission to hospital the malaria diagnosis was not delayed. This was due to a proactive case surveillance mechanism in operation by the AMC where PHFOs visit hospital wards daily and screen all patients with fever for malaria. Early diagnosis was particularly important in this patient in whom a further delay in treatment might have been life-threatening because of his co-morbidities. It reaffirms the important role of active surveillance for malaria during the POR phase.

This case is a reminder that the risk of transfusion-induced malaria in the country persists even after elimination because of imported malaria from overseas. It demonstrates that even the current very rigorous POR strategies being implemented in Sri Lanka are not infallible, due, not to flaws in the strategy itself, but due to lapses in operations and implementation. The blood donor was duly screened for malaria on entry to the country but was found to be negative by both microscopy and RDT. This could have been due to a very low parasite density, below the microscopic threshold of detection, which is corroborated by the fact that the person was asymptomatic and was even well enough to have considered donating blood 4 months later. There have been numerous incidents in the past 9 years since malaria elimination, of arrivals in Sri Lanka from malaria endemic countries being screened on entry to the country and found negative, but who, weeks to months later developed a clinically patent malaria infection, even with *P. falciparum* [14]. Clearly *Plasmodium vivax* and *Plasmodium ovale* infections can remain dormant as hypnozoites in the liver, but it appears that even *P. falciparum* parasites can persist in a “dormant” state in the blood to manifest as clinical infections later. The asymptomatic blood donor, by the fact that he was not even anaemic, whilst harbouring a *P. falciparum* infection over several months, must have developed a partial clinical and parasitological immunity to *P. falciparum* while in South Sudan through repeat parasite inoculations, aided by his intermittent recourse to prophylactic anti-malarial medicines which may have led to a persistent low-grade malaria infection.

*Plasmodium* parasites can survive in whole blood and plasma when stored at 4 °C for up to approximately 18 days, and parasites can be detectable even up to 28 days when frozen, although with diminished infectivity [15, 16]. Consistent with WHO recommendation and the National Institute of Health Consensus conference outcomes in 1995, every blood donor in Sri Lanka is screened for HIV, hepatitis B and C, syphilis and malaria, and for the latter by microscopic examination of Giemsa-stained thick blood smears. Although this blood donor was screened for malaria and a negative report was issued, the blood was found, retrospectively, on re-examination of the preserved smear to be malaria-infected. As has been reported previously, as much as 40% of approximately one million blood smears examined every year for malaria by the AMC are from the blood bank and not a single smear has been positive for the past 9 years; data for the years 2017–2019 have been published [2]. Given this large volume, and because the test positivity rate is extremely low, malaria being a very rare disease in the country now, microscopic screening of blood donors for malaria cannot be expected to have a high sensitivity even in the hands of a highly trained microscopists. Under these circumstances, other, automated and more objective methods of blood screening such as immunological assays (e.g. ELISAs based on antigen and antibody detection [17]) may have to be considered as alternatives to microscopy for screening of blood donors for malaria. A recent meta analysis of transfusion malaria data compares the sensitivities of microscopy, serological methods and molecular techniques [18] in screening of blood donors, and concludes that although molecular methods may provide the highest degree of sensitivity, given their high cost and low feasibility in some situations, a careful consideration of the circumstances and available resources will have to determine which of these methods should be deployed. This incident highlights the need to sustain a high-quality microscopy service, which can be quite challenging when the disease burden is as low as it is in Sri Lanka today. The country is, in fact, engaged in a WHO scheme on External Competency Assessment of Malaria Microscopy (ECAMM) and has one of the highest proportions of high quality (Level 1 and 2) malaria microscopists amongst countries in the region as assessed by this scheme [19]. A National Competency Assessment of Malaria Microscopy (NCAMM) scheme has also been established in the country, and these efforts are expected to increase the competencies of malaria microscopists more widely across the country. Following this event of transfusion-induced malaria, a more stringent policy has also now been adopted by the national blood bank to prevent such events in the future: The period during which persons who have returned from a malaria endemic country are not permitted to donate blood has been extended from the previous policy of 3 months, to 3 years from their return.

The blood donor, a member of the armed forces and a returnee from South Sudan was duly screened for malaria at the airport on his return together with 51 members of the same contingent, and all were negative on microscopy and RDT. It is standard practice that all such groups of armed service personnel returnees are kept under surveillance for a period of one year for the possibility of developing malaria. If they reside in a malaria receptive area, they are each provided with a LLIN as a precautionary measure to minimize the risk of their transmitting malaria, in the event that they develop an asymptomatic malaria infection. He was traced on the AMC database even before details emerged from the Blood Bank, as a member of the group that was under surveillance. However, a lapse in the follow-up procedure led to this person who was asymptomatic, to donate blood. The prevailing COVID 19 epidemic which led to several restrictions being imposed on routine follow-up work of the AMC might have contributed to this unfortunate breach in procedure [20]. The fact that he was parasitaemic with mature gametocytes in the circulation *albeit* at a very low parasitaemia for at least 18 days, and possibly longer, had presented a daunting risk for the re-establishment of malaria in Sri Lanka had his infection been transmitted to mosquitoes. This case has now led to the AMC greatly strengthening its surveillance on armed forces returnees from malaria endemic countries, they being a high-risk group for imported malaria including considering the routine use of more sensitive malaria detection methods than microscopy, such as the polymerase chain reaction tests to screen returnees from high malaria risk destinations.

This case report illustrates the importance of effective blood donor screening for malaria during the POR phase of malaria using more sensitive and objective methods than microscopy, such as antigen and antibody detecting immunochemical methods. It also reaffirms the importance of proactive case detection and follow-up, particularly in armed forces personnel returning from high malaria risk destinations, here too, using molecularly methods rather than microscopy, and emphasizes the need for stringent implementation of the strategies that are in place including reactive entomological surveillance and vector control.

## Conclusion

Transfusion-induced malaria is among the risk factors for re-establishment of malaria after elimination. Screening of blood donors for malaria by microscopy can be low in sensitivity even in the hands of expert microscopists because the test positivity rate is extremely low. Population groups who are at high risk of imported malaria such as armed forces personnel returning from malarious countries must be screened using more sensitive methods than microscopy and kept under close surveillance for at least a year, for the possibility of their developing malaria infections, even if they are negative for malaria on arrival in the country.

## Supplementary Information

**Additional file 1: Table S1.** Data on beta thalassaemia patient diagnosed with malaria.

## Data Availability

The datasets generated and/or analysed in this publication are not publicly available due to the fact that they belong to the Ministry of Health, Sri Lanka. Clarifications regarding data can be made through Dr. Prasad Ranaweera, Director of the Anti Malaria Campaign, Sri Lanka who is an author of this publication.

## Abbreviations

ACT: Artemisinin-based combination therapy
ALT: Alanine transaminase
AMC: Anti Malaria Campaign
AST: Aspartate Transaminase
DHAPPQ: Dihydroartemisinin-piperaquine
ELISA: Enzyme linked immunosorbent assay
HIV: Human Immunodeficiency Virus
LLIN: Long lasting impregnated nets
NCAMM: National Competency Assessment of Malaria Microscopy
PHFO: Public Health Field Officer
POR: Prevention of re-establishment
RDT: Rapid Diagnostic Tests
WBC: White Blood Cells
WHO: World Health Organization
