# Supplementary material for: Transfusion-induced Plasmodium falciparum malaria in a beta thalassaemia patient during the prevention of re-establishment phase in Sri Lanka
Source: Malar J. 2021 Aug 26;20:352. doi: 10.1186/s12936-021-03881-1 (PMC8390059; doi:10.1186/s12936-021-03881-1)
Supplement: Supplementary file 1 — Additional file 1: Table S1. Data on beta thalassaemia patient diagnosed with malaria. [file 12936_2021_3881_MOESM1_ESM.docx]

**Table S1. Clinical management data on the beta thalassemia malaria patient**

| Date | Day since Rx** | Period of day | Malaria parasite density (parasites/µl) | | FBC (mg/L) | | | Liver function tests (U/L) | | CRP (mg/L) | Renal function | Malaria infection milestones |
| --- | --- | --- | --- | --- | --- | --- | --- | --- | --- | --- | --- | --- |
|  |  |  | **Rings** | **G’cytes*** | **WBC(*1000)** | **Hb** | **Platelets (*1000)** | **AST** | **ALT** |  | **S. Creatinine** |  |
| 09/05/21 |  |  | 46,327 | 4988 | 24.7 | 5.6 | 347 | 44.2 | 44.8 | 132 | 43 | Both pLDH/ HRP2 RDT, & microscopy positive |
| 10/05/21 | D0 | Morning | 33,579 | 8833 | 31.3 | 5.4# | 378 | ND | ND | ND | 42.8 | Oral ACT started |
|  |  | Evening | 10,331 | 9244 |  |  |  |  |  |  |  |  |
| 11/05/21 | D1 | Morning | 9,225 | 8289 | 32.1 | 7.1 |  | 86.5 | 62 | 142 |  |  |
|  |  | Evening | 8,343 | 7442 |  |  |  |  |  |  |  |  |
| 12/05/21 | D2 | Morning | 6,184 | 7369 | 48.2 | 8.4 | 348 | 250.8 | 174.3 | 133 | 34.7 |  |
|  |  | Evening | 3,639 | 3952 |  |  |  |  |  |  |  |  |
| 13/05/21 | D3 | Morning | 2269 | 5300 | 47.4 | 7.6 | 276 | 458.9 | 314.9 | 111 | 33.6 | Oral ACT completed  IV artesunate started |
|  |  | Evening | 4236 | 2911 |  |  |  |  |  |  |  |  |
| 14/05/21 | D4 | Morning | 3922 | 2888 | 41.4 | 8.4 | 348 | 264.3 | 279 | 70.6 | 34.5 |  |
|  |  | Evening | 1738 | 3122 |  |  |  |  |  |  |  |  |
| 15/05/21 | D5 | Morning | 541 | 2214 | 23.8 | 7.7 | 362 | 11.2 | 194.4 | 38.3 | 33.1 |  |
|  |  | Evening | 181 | 1645 |  |  |  |  |  |  |  |  |
| 16/05/21 | D6 | Morning | 182 | 1589 | 15.6 | 7.4 | 165 | 52 | 145.6 | ND | 33.3 |  |
|  |  | Evening | 61 | 685 |  |  |  |  |  |  |  |  |
| 17/05/21 | D7 | Morning | 0 | 270 | 19.9 | 6.9 | 494 | 48.7 | 25.5 | 43.2 | ND | IV Artesunate completed. Oral ACT restarted |
|  |  | Evening | 0 | 258 |  |  |  |  |  |  |  |  |
| 18/05/21 | D8 | Morning | 0 | 257 | 38.3 | 6.9## | 586 | 53.7 | 99.0 | ND | 35.8 |  |
|  |  | Evening | 0 | 135 |  |  |  |  |  |  |  |  |
| 19/05/21 | D9 | Morning | 0 | 140 | 25.3 | 8.7 | 671 | 58.7 | 81.5 | ND | 46.5 | Oral ACT completed |
|  |  | Evening | 0 | 152 |  |  |  |  |  |  |  |  |
| 20/05/21 | D10 | Morning | 0 | 62 | 17.2 | 8.0 | 666 | 79.4 | 76.1 | ND | ND | PQ stat dose given  (7.5 mg x 3 tabs) |
|  |  | Evening | 0 | 61 |  |  |  |  |  |  |  |  |
| 21/05/21 | D11 | Morning | 0 | 67 | 17.6 | 8.0 | 631 | 111.8 | 88.3 | ND | ND |  |
|  |  | Evening | 0 | 34 |  |  |  |  |  |  |  |  |
| 22/05/21 | D12 | Morning | 0 | 40 | 21.3 | 8.3 | 682 | ND | ND | 19.5 | 34.9 |  |
|  |  | Evening | 0 | 38 |  |  |  |  |  |  |  |  |
| 23/05/21 | D13 | Morning | 0 | 44 | 23.9 | 7.3 | 617 | 168.6 | 125.4 | ND | ND | RDT- HRP2 only positive |
|  |  | Evening | 0 | 44 |  |  |  |  |  |  |  |  |
| 24/05/21 | D14 | Morning | 0 | 46 | 24.7 | 7.1 |  | 177.4 | 140.2 | ND | 37.1 |  |
|  |  | Evening | 0 | 49 |  |  |  |  |  |  |  |  |
| 25/05/21 | D15 | Morning | 0 | 0 | 26.6 | 6.6 | 577 | ND | ND | ND | ND |  |
|  |  | Evening | 0 | 0 |  |  |  |  |  |  |  |  |
| 26/05/21 | D16 | Morning | 0 | 0 | 22.5 | 6.6### | 567 | 255.8 | 209.6 | ND | ND |  |
|  |  | Evening | 0 | 0 |  |  |  |  |  |  |  |  |
| 27/05/21 | D17 |  | 0 | 0 | 17.27 | 9.8 | 524 | 313.1 | 265.5 | ND | 36.8 |  |

** Rx – treatment; * Gametocytes ; FBC- Full Blood Count; WBC- White Blood Count ; Hb- hemoglobin; AST- Aspartate Aminotransferase; ALT- Alanine Transferase;

CRP- C Reactive Protein ; pLDH- Parasite Lactase Dehydrogenase; HRP2- Histidine-Rich Protein 2; ACT- artemisinin-based combination therapy; RDT- Rapid Diagnostic Test; PQ- Primaquine; ND- Not done

# 1^st^ transfusion on 10/05/21- Pre Tx Hb 5.4, post Tx Hb 7.1

## 2^nd^ Tx on 18/05/2021- Pre Tx Hb ,  6.9   post Tx Hb 8.7

### 3^rd^ Tx 26/05/21- Pre Tx Hb 6.6, Post Tx Hb 9.8
